# Efficacy of prolotherapy in temporomandibular joint disorders with hypertonic dextrose and Polydeoxyribonucleotide (PDRN)

**DOI:** 10.22514/jofph.2025.062

**Published:** 2026-01-12

**Authors:** Jin-Won Choi, Young-Kyun Kim, Pil-Young Yun, Jeong-Kui Ku

**Affiliations:** ^1^Department of Oral and Maxillofacial Surgery, Section of Dentistry, Seoul National University Bundang Hospital, 13620 Seongnam-si, Republic of Korea; ^2^K Oral and Maxillofacial Surgery Dental Clinic, 13637 Seongnam-si, Republic of Korea; ^3^Department of Dentistry and Dental Research Institute, School of Dentistry, Seoul National University, 03080 Seoul, Republic of Korea

**Keywords:** Temporomandibular joint disorder, Prolotherapy, Hypertonic dextrose, Polydeoxyribonucleotide (PDRN), Pain management

## Abstract

**Background**: This study evaluated the clinical efficacy of prolotherapy using hypertonic dextrose and polydeoxyribonucleotide (PDRN) in patients with temporomandibular joint disorders (TMDs) who did not respond to conventional treatments. **Methods**: A retrospective chart review of 66 patients diagnosed with TMD was conducted. Patients underwent prolotherapy between March and December 2024 and were classified into soft-tissue-related and bone-related TMD groups. Treatment involved injections of hypertonic dextrose or PDRN targeting anatomical structures within the temporomandibular joint (TMJ). Pain and function were assessed using the visual analog scale (VAS) and maximum mouth opening (MMO). Additional parameters, including joint sounds and jaw displacement (S deviation or L deflection), were analyzed. Outcomes were measured at baseline, after each prolotherapy session, and during the final follow-up. Statistical analyses included paired *t*-tests, McNemar’s tests, Analysis of Variance (ANOVA), and regression modeling. **Results**: Prolotherapy was administered an average of 2.3 times per patient. The baseline VAS score decreased from 4.34 ± 2.12 to 1.00 ± 1.58 (*p * < 0.001), and MMO improved from 31.0 ± 8.7 mm to 40.8 ± 4.55 mm (*p * < 0.001). Joint sounds, jaw displacement, and deflection also showed significant reductions. Comparative analysis of prolotherapy agents revealed no statistically significant differences between the PDRN and dextrose groups, although both demonstrated significant improvements in MMO and VAS scores. Among patients with baseline joint sounds, 23 individuals experienced complete resolution of sounds, along with significant reductions in jaw displacement (69.6% to 13.0%, *p * < 0.001) and deflection (52.2% to 8.7%, *p * < 0.001). **Conclusions**: Prolotherapy is an effective intervention for improving pain and jaw function in TMD patients. Both hypertonic dextrose and PDRN demonstrated significant clinical improvements on TMD prolotherapy.

## 1. Introduction

The temporomandibular joint (TMJ) is a unique anatomical structure that connects the mandible to the skull. It possesses several distinctive features, including its ability to move in three dimensions, its role in load-bearing, and its direct relationship with dental occlusion [1]. Disorders of the TMJ, collectively termed temporomandibular joint disorders (TMDs), are multifactorial in origin. These include neuromuscular dysfunction, developmental abnormalities, parafunctional habits, trauma, nutritional deficiencies, hormonal imbalances, and metabolic disturbances [2]. Primary conservative treatments for TMD management typically include nonsteroidal anti-inflammatory drugs (NSAIDs) for pain and inflammation control, occlusal splint therapy, and physical therapy. If conservative measures fail, more invasive approaches such as trigger point injections with botulinum toxin or lidocaine, intra-articular injections of steroids or hyaluronic acid, lavage, and even open surgical procedures may be considered. Traditionally, these treatment strategies have focused primarily on major structures, such as the masticatory muscles and bone [3]. However, recent evidence suggests that the etiology of TMD may also involve inflammatory changes and chronic pain within minor anatomical structures, including the articular disc, retrodiscal tissues, TMJ-associated ligaments and capsules, and tendons. Conventional approaches offer limited options for addressing these minor structures, necessitating the exploration of novel interventions.

In recent years, prolotherapy has emerged as a promising treatment alternative for TMD. Prolotherapy is defined as a non-surgical regenerative therapy designed to stimulate cellular proliferation and restore the integrity of weakened ligaments and tendons [3]. Also known as “regenerative injection therapy”, prolotherapy aims to promote the growth and proliferation of new, healthy connective tissue. When applied to the TMJ, prolotherapy targets ligaments, tendons, and other supportive structures around the joint [4, 5]. The most commonly used proliferant is hypertonic dextrose, but other agents such as polidocanol, manganese, zinc, human growth hormone, and platelet-rich plasma (PRP) have also been explored. In the orthopedic field, polydeoxyribonucleotide (PDRN) has shown promise as an effective proliferant [6]. PDRN is a mixture of deoxyribonucleotide polymers, typically extracted from salmon sperm, with chain lengths ranging from 50 to 2000 base pairs. Its therapeutic effects are attributed to its ability to reduce inflammation by downregulating pro-inflammatory mediators such as tumor necrosis factor-alpha (TNF-α), interleukin-6 (IL-6), and high-mobility group box 1 (HMGB1), and stimulate tissue repair and wound healing by upregulating vascular endothelial growth factor (VEGF) expression [7, 8]. Despite its promising applications in regenerative medicine, the mechanisms of prolotherapy in TMJ treatment remain poorly understood. Furthermore, studies evaluating the efficacy and indications of various proliferants, including dextrose and PDRN, for TMJ disorders are still limited.

TMJ prolotherapy offers a minimally invasive approach that may benefit patients with TMD who fail to respond to conservative therapies for more than three months, prefer to avoid surgical interventions, and experience symptoms arising from minor anatomical structures within the TMJ [9]. The primary aim of this study is to evaluate the efficacy of prolotherapy in improving mouth opening capacity, pain levels, and joint sounds in patients with TMD who have shown no response to conservative treatments for a period of three months or longer. As a secondary objective, this study aimed to compare two agents with distinct mechanisms. We selected hypertonic dextrose, the most traditional agent that functions via a controlled inflammatory response, and PDRN, a newer, non-inflammatory agent that promotes tissue regeneration. The primary hypothesis was that TMJ prolotherapy would significantly reduce pain and improve mouth opening in this treatment-resistant patient population. A secondary hypothesis was that there would be no significant difference in clinical efficacy between the two agents.

## 2. Materials and methods

All procedures in this study adhered to the ethical guidelines set by the institutional and national committees responsible for human experimentation, in accordance with the Helsinki Declaration of 1975, revised in 2008. This retrospective study was approved by the Institutional Review Board of Seoul National University Bundang Hospital (IRB No. B-2501-948-103). It focused on patients diagnosed with TMD who received prolotherapy treatment in the TMJ region between March 2024 and December 2024. Data for this retrospective study were extracted from a single institution’s standardized electronic medical record (EMR) system. To ensure consistency, only charts with complete data for the primary outcomes (VAS and MMO) at the pre-defined assessment points were included in the final analysis. The diagnosis of TMD for all included patients was based on a comprehensive evaluation, which included a clinical examination, a review of patient history, and standard radiological imaging. The inclusion criteria required adult patients experiencing TMD-related pain that persisted for at least three months despite conventional treatments such as supported self-management, physical therapy, splint therapy, botulinum toxin injections into the masseter or temporalis muscles, intra-articular injections of dexamethasone or hyaluronic acid, and arthrocentesis or TMJ arthroscopy [10]. Patients were also required to have voluntarily completed a Visual Analog Scale (VAS) questionnaire assessing TMJ-related pain. The exclusion criteria eliminated patients diagnosed with diabetes mellitus, those with TMJ tumors or bony ankylosis, individuals with a history of TMJ open surgery, patients taking NSAIDs or corticosteroids for systemic disease management, individuals requiring psychiatric treatment, and patients with asymptomatic TMJ sounds as their sole symptom. Due to the limitations of a retrospective chart review, a standardized diagnostic system such as the Diagnostic Criteria for Temporomandibular Disorders (DC/TMD) could not be retroactively applied. Therefore, patients were classified into two broad categories based on their primary documented symptoms and findings: bone-related TMD, comprising patients diagnosed solely with osteoarthritis; and soft-tissue-related TMD, a heterogeneous group that included disorders of the masticatory muscles, ligaments, capsule, or articular disc. This classification was used to explore potential differences in treatment outcomes in the subsequent analysis.

### 2.1 Prolotherapy protocol

The prolotherapy procedure was conducted in accordance with previously established protocols [11]. Initially, for pain control, an auriculotemporal nerve block and superior joint space infiltration anesthesia was administered using 1.8 mL mepivacaine hydrochloride or 1.8 mL of 2% lidocaine with 1:100,000 epinephrine [12]. Prolotherapy utilized either a hypertonic dextrose solution or Polydeoxyribonucleotide (PDRN, Placentex®, Rejuvenex®, PolyNeo®) [13]. Group allocation to either the hypertonic dextrose or PDRN group was based on the patient’s choice after explanation of the two options. Based on prior studies on dextrose injection concentrations, a 10% dextrose solution was prepared by mixing 20% dextrose (4 g/20 mL), 2% lidocaine, and normal saline (180 mg/20 mL) in a 2:1:1 ratio [14, 15]. The treatment interval was determined by the agent used, based on established protocols. Injections of the hypertonic dextrose solution were administered approximately every three weeks. PDRN injections were administered at 1- to 2-week intervals. The anatomical targets for TMJ prolotherapy included posterior disc attachment tissues, anterior disc attachment tissues, the superior portion of the lateral capsule, and the inferior portion of the lateral capsule. At each targeted anatomical site, either the hypertonic dextrose solution or PDRN was injected in volumes of 2 cc to the posterior disc attachment tissues, 1 cc to the anterior disc attachment, and 0.5 cc to the superior and inferior portion of lateral ligament. For pain control, patients were prescribed aceclofenac 100 mg once a day for 7 days or ibuprofen 200 mg three times a day for 2 days. Other interventions were discontinued during the prolotherapy treatment and follow-up period. However, patients who had previously been fitted with splints were instructed to continue using them throughout the treatment. This decision was made to mitigate the potential negative impact of uncontrolled nocturnal parafunctional habits like bruxism on the therapeutic outcomes.

### 2.2 Clinical evaluation

To minimize observer bias, the retrospective clinical evaluation was performed by an independent evaluator who was not involved in patient treatment. This evaluator was blinded to the type of prolotherapy agent administered to each patient while extracting and analyzing outcome data, such as VAS and MMO scores, from the clinical charts. The severity of pain related with TMD was assessed using a VAS ranging from 0 to 10, where 10 represented the “most severe pain or functional impairment imaginable” [16]. MMO was measured as the distance between the maxillary and mandibular central incisors, plus the vertical overlap (overbite) to reflect the true range of motion. All patients included in the study exhibited trismus (MMO <35 mm) that was refractory to previous treatments. Additional evaluations included TMJ sounds (clicking, popping, and crepitus) and jaw displacement during opening, characterized by S-shaped deviations or ipsilateral deflections. These symptoms were periodically measured at each session to compare differences before and after prolotherapy treatment. The interval between prolotherapy sessions was typically 2–3 weeks. At each visit, the patient’s symptoms were evaluated to determine if further injections were required. If TMD symptoms persisted, additional prolotherapy injections were administered. For bilateral TMD cases, treatment continued until symptoms in both joints were resolved. Clinical outcomes were analyzed on a per-patient basis rather than by symptomatic joint. The final clinical outcomes were assessed at a separate post-treatment follow-up visit.

The analysis incorporated various statistical methods to assess the effects of prolotherapy treatment. Descriptive statistics, including mean and standard deviation, were used to summarize data distribution and trends. A one-way Analysis of Variance (ANOVA) was conducted to assess group differences based on TMD classifications, including soft tissue-related disorders (masticatory muscle, ligament, or articular disc) and osteoarthritis. Paired *t*-tests were employed to evaluate pre- and post-treatment differences in pain (VAS) and MMO. Changes in continuous and categorical variables before and after treatment were analyzed using paired *t*-tests and McNemar’s tests, respectively. Group differences were evaluated with independent *t*-tests and Chi-square tests. Descriptive statistics, including mean and standard deviation, were calculated for MMO and VAS at both time points. Pearson’s correlation coefficient (*r*) and *p*-values were computed to evaluate linear relationships between the two variables. Linear regression modeling was applied to assess whether MMO could predict VAS values. Multivariable regression analysis investigated the interaction effects of injection counts, clicking, and deviation on symptom changes, providing insights into the combined influence of multiple factors. All statistical analyses were performed using R version 4.2.20, with a significance threshold set at *p * < 0.05.

## 3. Results

A total of 66 patients (14 males and 52 females, with a mean age of 45.6 ± 16.5 years) were included in this study, as summarized in Table 1. The participants experienced TMD symptoms for an average duration of 24.9 months. Of the total sample, 51 patients were diagnosed with soft tissue-related problems, including disorders affecting the masticatory muscles, ligaments, and articular disc. The remaining 15 patients were identified as having osteoarthritis of the TMJ without any accompanying soft tissue issues (Fig. 1).

**Table 1.** Baseline characteristics and prolotherapy treatment summary.

Variables	Values		
Age (yr)	45.6 ± 16.5		
Male:Female	14:52		
Unilateral:Bilateral TMD	57:9		
Numbers of prolotherapy	N (%)		
• 1	15 (22.7%)		
• 2	6 (9.1%)		
• 3	34 (51.5%)		
• 4	9 (13.6%)		
• 6	1 (1.5%)		
• 7	0 (0.0%)		
• 8	1 (1.5%)		
Averaged follow-up period (mon)	2.4 ± 1.0		
	Baseline	Final follow-up	*p*-value
MMO (mm)	31.00 ± 8.69	40.82 ± 4.55	<0.001*
Pain score (VAS)	4.34 ± 2.12	1.00 ± 1.58	<0.001*
Joint sound	59.1% (n = 39)	36.4% (n = 24)	0.015^¶^
Jaw displacement	57.6% (n = 38)	22.7% (n = 15)	<0.001^¶^
Jaw deflection	42.4% (n = 28)	18.2% (n = 12)	0.005^¶^

*: Paired *t*-test. ^¶^: McNemar’s test. TMD, temporomandibular disorders; MMO, maximum mouth opening; VAS, visual analog scale.

**Fig. 1.** Classification of temporomandibular disorders (TMD) and symptom distribution.

The baseline characteristics of TMD symptoms included a mean pain score of 4.34 ± 2.12 on the VAS and a mean MMO of 31.0 ± 8.7 mm. Joint sounds, such as clicking, crepitus, and popping, were observed in 59.1% of patients, while jaw displacement, including S-shaped deviation and deflection during opening, was reported in 57.6% of cases. Additionally, 42.4% of patients exhibited deflection during mouth opening. Prolotherapy was administered an average of 2.3 ± 0.7 times per patient. Most patients (83.3%) received prolotherapy within three sessions. Clinical outcomes were evaluated after up to three sessions and during the final follow-up period, which occurred 2.4 ± 1.0 months post-treatment (Table 1). Significant improvements were observed in both MMO and VAS scores. Pain scores (VAS) decreased from 4.34 to 1.00, while MMO increased from 31.0 mm to 40.8 mm. These changes were statistically significant, with *p*-values < 0.001 (Fig. 2).

**Fig. 2.** Changes in VAS and MMO following prolotherapy treatment. VAS, visual analog scale; MMO, maximum mouth opening.

The baseline mean VAS was 4.34 ± 2.12, which showed a decrease to 1.68 ± 1.92 after the first session (*p * < 0.001). The second session recorded a slight increase to 2.65 ± 2.55 (*p* = 0.117), followed by a decrease to 2.08 ± 2.16 after the third session (*p* = 0.325). The final evaluation demonstrated a significant reduction to 1.00 ± 1.58 (*p * < 0.001), indicating effective pain management over the treatment course. The baseline mean MMO was 31.00 ± 8.69 mm, which significantly increased to 37.31 ± 5.40 mm after the first session (*p * < 0.001). Further increases were observed in subsequent sessions, reaching 38.60 ± 5.62 mm (*p* = 0.066) and 39.47 ± 5.11 mm (*p* = 0.259) by the third session. The final MMO measurement showed a stable improvement to 40.82 ± 4.55 mm (*p* = 0.221).

Other TMD symptoms also improved significantly. The percentage of patients with joint sounds (*e.g.*, clicking, popping, or crepitus) decreased from 59.1% to 36.4% (*p* = 0.015). Jaw displacement, including S-shaped deviations and deflections, showed a significant reduction from 57.6% to 22.7% (*p * < 0.001). Similarly, deflection during mouth opening decreased from 42.4% to 18.2% (*p* = 0.005).

Pearson’s correlation analysis revealed a weak negative correlation (*r* = −0.26) between MMO and VAS, with a *p*-value of 0.0327, indicating that the relationship was statistically significant despite its relatively low strength. Linear regression analysis further examined this relationship, yielding a slope (β) of −0.26 (*p* = 0.033) and an *R*^2^ value of 0.068, indicating a statistically significant relationship, albeit with a relatively low explanatory power (Fig. 3).

**Fig. 3.** Scatter plot showing the relationship between maximum mouth opening (MMO) and pain score (VAS) after prolotherapy.

Regression analysis was performed to investigate factors influencing changes in MMO and VAS. For MMO change, the model yielded an *R*^2^ value of 0.071, indicating that the independent variables explained 7.1% of the variance. However, none of the predictors were statistically significant. These predictors included sex (*p* = 0.892), side of involvement (unilateral or bilateral, *p* = 0.600), TMD subtypes (only osteoarthritis without soft tissue problems or other TMDs with soft tissue problems, *p* = 0.187; only soft tissue problems or soft tissue problems with osteoarthritis, *p* = 0.424; TMD symptoms with or without masticatory muscle problems, *p* = 0.610), use of stabilization splint therapy (*p* = 0.567), baseline joint sound (*p* = 0.194), and baseline deviation or deflection during mouth opening (*p* = 0.708).

For VAS changes, the model yielded an *R*^2^ value of 0.168, explaining 16.8% of the variance. Among the predictors, the side of involvement (*p* = 0.088) and splint therapy (*p* = 0.092) showed borderline significance. Other predictors, including sex (*p* = 0.868), TMD subtypes (only osteoarthritis without soft tissue problems or other TMDs with soft tissue problems, *p* = 0.230; only soft tissue problems or soft tissue problems with osteoarthritis, *p* = 0.124; TMD symptoms with or without masticatory muscle problems, *p* = 0.353), baseline joint sound (*p* = 0.627), and baseline deviation or deflection during mouth opening (*p* = 0.198), did not reach statistical significance.

### 3.1 Comparison of treatment outcomes between PDRN and hypertonic dextrose solution in TMJ prolotherapy

In comparison with prolotherapy agents between PDRN and hypertonic dextrose solution, the baseline characteristics, including age, sex, TMD side, and baseline MMO and VAS, showed no significant differences between the two groups (Table 2). In the PDRN group, the MMO significantly increased from a baseline value of 29.13 ± 7.34 mm to a final value of 40.00 ± 3.68 mm (*p * < 0.001). VAS also showed a significant reduction, decreasing from 4.70 ± 1.78 to 0.98 ± 1.21 (*p * < 0.001). Similarly, in the hypertonic dextrose solution group, the MMO improved significantly from 32.56 ± 9.49 mm at baseline to 41.50 ± 5.12 mm at the final follow-up (*p * < 0.001). VAS decreased significantly from 4.04 ± 2.35 to 1.01 ± 1.84 (*p * < 0.001). Both groups demonstrated significant improvements in MMO and VAS scores after prolotherapy treatment (Fig. 4). However, no statistically significant differences were observed between the two groups in terms of treatment outcomes.

**Table 2.** Baseline characteristics and treatment outcomes of PDRN and hypertonic dextrose solution groups in TMJ prolotherapy.

		PDRN (n = 30)	Dextrose (n = 36)	*p*-value
Age (yr)		46.6 ± 17.4	44.8 ± 16.0	0.664*
Male:Female		6:24	8:28	>0.999^¶^
Unilateral:Bilateral TMD		26:4	31:5	0.667^¶^
MMO				
	Baseline	29.13 ± 7.34	32.56 ± 9.49	0.104*
	Final follow-up	40.00 ± 3.68	41.50 ± 5.12	0.172*
VAS				
	Baseline	4.70 ± 1.78	4.04 ± 2.35	0.201*
	Final follow-up	0.98 ± 1.21	1.01 ± 1.84	0.936*
Joint sound				
	Baseline	53.3% (n = 16)	63.9% (n = 23)	0.537^¶^
	Final follow-up	36.7% (n = 11)	36.1% (n = 13)	>0.999^¶^
Jaw displacement				
	Baseline	66.7% (n = 20)	50.0% (n = 18)	0.265^¶^
	Final follow-up	26.7% (n = 8)	22.2% (n = 8)	>0.999^¶^
Jaw deflection				
	Baseline	50.0% (n = 15)	36.1% (n = 13)	0.375^¶^
	Final follow-up	20.0% (n = 6)	16.7% (n = 6)	0.977^¶^

*: Independent *t*-test. ^¶^: Chi-square test. TMJ, temporomandibular joint; PDRN, polydeoxyribonucleotide; TMD, temporomandibular disorders; MMO, maximum mouth opening; VAS, visual analog scale.

**Fig. 4.** Comparison of MMO and VAS changes between PDRN and hypertonic dextrose solution groups in TMJ prolotherapy. MMO, maximum mouth opening; VAS, visual analog scale; PDRN, polydeoxyribonucleotide.

### 3.2 Clinical outcomes for patients with resolved joint sounds after prolotherapy

Among the 39 patients who initially presented with joint sounds at baseline, a total of 23 individuals (mean age 44.7 ± 19.1 years; 5 males and 18 females) demonstrated complete resolution of joint sounds following prolotherapy treatment. The mean MMO increased from 33.70 mm to 41.04 mm, while the VAS for pain decreased from 4.21 to 0.59. Additionally, the prevalence of jaw displacement was reduced from 69.6% to 13.0%, and jaw deflection during mouth opening decreased from 52.2% to 8.7%. These findings indicate substantial clinical improvements in jaw function, pain relief, and symptom resolution in patients who experienced the disappearance of joint sounds after treatment (Table 3).

**Table 3.** Changes in clinical parameters for patients with resolved joint sounds after prolotherapy treatment.

	Baseline	Final follow-up	*p*-value
MMO (mm)	33.70 ± 8.28	41.04 ± 4.55	<0.001*
Pain score (VAS)	4.21 ± 1.98	0.59 ± 0.78	<0.001*
Jaw displacement	69.6% (n = 16)	13.0% (n = 3)	<0.001^¶^
Jaw deflection	52.2% (n = 12)	8.7% (n = 2)	<0.001^¶^

*: Paired *t*-test. ^¶^: McNemar’s test. MMO, maximum mouth opening; VAS, visual analog scale.

## 4. Discussion

Prolotherapy has been recognized since the 1930s for its ability to enhance tendon, ligament, and joint stabilization [4]. However, its effects on the TMJ have remained largely unexplored. This study highlights the clinical significance of prolotherapy as a promising alternative treatment for patients with TMD who fail to respond to conventional therapies. The findings of this study supported our primary hypothesis that TMJ prolotherapy is an effective intervention for improving pain and jaw function in patients with refractory TMD. Furthermore, our secondary hypothesis was also supported, as no statistically significant difference in clinical efficacy was found between the hypertonic dextrose and PDRN groups. Both hypertonic dextrose and PDRN demonstrated significant improvements in pain relief, jaw function, and symptom resolution, including reductions in joint sounds, jaw displacement, and deflection (Table 1). Prolotherapy effectively increased MMO and reduced pain scores (VAS), with comparable outcomes observed across different TMD subtypes and treatment agents. Moreover, a statistically significant correlation was found between MMO and VAS, further supporting its therapeutic efficacy.

Prolotherapy is widely used in orthopedic and musculoskeletal treatments, including the knee, finger, and lumbar regions, yielding favorable results [17, 18, 19]. While the exact mechanism of prolotherapy in TMD remains unclear, it is believed that the injection of proliferative agents stimulates fibroblast proliferation through both inflammatory and non-inflammatory pathways, thereby strengthening weakened or inflamed connective tissues and reducing pain [20]. Fibroblasts promote angiogenesis, cell proliferation, and collagen deposition, activating essential growth factors such as platelet-derived growth factor, transforming growth factor-β, connective tissue growth factor, and epidermal growth factor [21]. This dynamic tissue remodeling process is particularly suited to treating soft tissue structures such as tendons, ligaments, joint capsules, and muscles, which are often difficult to manage with conventional TMD treatments. As such, prolotherapy has emerged as a viable alternative for chronic TMD patients who are unresponsive to conservative treatments like oral appliances or medications [22]. Interestingly, patients diagnosed solely with osteoarthritis also experienced significant improvements in MMO and VAS. A 2023 systematic review reported that dextrose prolotherapy demonstrated potential benefits in reducing pain and improving function in orthopedic cases of osteoarthritis [23]. Further studies are warranted to investigate whether similar improvements in pain and functional outcomes observed in the TMJ could also have protective effects against joint degradation.

Within this field, the therapeutic effect of prolotherapy is delivered through various types of proliferants. Traditional agents, like the hypertonic dextrose used in this study, are thought to work by inducing a localized inflammatory response [24]. A more modern category is orthobiologics, with PRP being a prominent example. However, the clinical application of PRP involves practical challenges, including the need for blood withdrawal and centrifugation, a lack of standardization, and potentially higher costs [25]. These limitations highlight the clinical need for an effective regenerative agent that is both standardized and readily available. PDRN is a standardized biopharmaceutical that meets these criteria, promoting tissue repair through a distinct, non-inflammatory mechanism via adenosine receptor agonism [13]. Therefore, this study was designed to compare two major therapeutic models: the traditional, inflammation-mediated approach of dextrose, and the standardized regenerative pharmacology offered by PDRN.

Hypertonic dextrose is used at a concentration of 10%, which induces cell wall lysis and fibroblast activation to initiate the regenerative process [14, 15, 26]. However, NSAIDs should be avoided, as they may interfere with the controlled inflammatory response required for effective prolotherapy. Although hypertonic dextrose is generally safe, it should be avoided in patients with uncontrolled or severe diabetes. In contrast, PDRN serves as a source of pyrimidines and purines, stimulating nucleic acid synthesis through salvage pathways [7, 8]. PDRN has been shown to reduce inflammatory mediators such as tumor necrosis factor-alpha (TNF-α), interleukin-1 (IL-1), interleukin-6 (IL-6), and high-mobility group box 1 (HMGB1) while promoting tissue repair through adenosine receptor (A2A) activation [6]. The positive effects of PDRN are attributed to this receptor activation, as studies have demonstrated that co-administration of PDRN with specific A2A receptor antagonists, such as 3,7-dimethyl-propargylxanthine, blocks its regenerative pathway [27, 28]. These findings support the potential of PDRN as a viable proliferative agent in prolotherapy for regenerative purposes. PDRN’s role as an effective alternative for arthritis treatment has been highlighted in previous studies, showing that it improves clinical symptoms, reduces histological damage, and decreases inflammatory cytokine production in stimulated human chondrocytes [28, 29]. Unlike dextrose prolotherapy, which relies on an initial inflammatory phase that can be suppressed by corticosteroids or NSAIDs, PDRN stimulates tissue proliferation without inducing inflammation, allowing for greater flexibility in medication use [6]. Patients undergoing dextrose prolotherapy often report transient, intense pain for several days due to the initial inflammatory response that promotes healing [30]. On the other hands, pain exacerbation following PDRN injections is rare, making it more tolerable for patients. Additionally, dextrose prolotherapy typically requires a minimum 3-week interval between treatments, resulting in a longer treatment course. On the other hand, PDRN injections are generally administered at 1- to 2-week intervals, offering a shorter treatment timeline and improved patient compliance compared to dextrose. In this study, both 10% hypertonic dextrose and PDRN demonstrated similar clinical efficacy in improving MMO and VAS scores (Table 2 and Fig. 4). Given the comparable outcomes, the choice of proliferative agent should consider factors such as injection intervals, patient tolerance to post-injection pain, and treatment costs. These findings suggest that prolotherapy, regardless of the proliferative agent used, holds promise as an effective treatment for soft tissue healing in patients with chronic TMD.

Recent trends in TMJ injection therapy underscore the importance of diversifying therapeutic agents. A mapping review by Chęciński *et al*. [31] clearly illustrated a growing research interest in finding new regenerative alternatives to traditional steroids or hyaluronic acid therapy. Our study makes a meaningful contribution to this trend of exploring alternative and regenerative treatments. The hypertonic dextrose and PDRN evaluated in this study are prime examples of such alternative options. PDRN is a promising agent with a novel, non-inflammatory mechanism for tissue regeneration. Simultaneously, hypertonic dextrose, though established in other orthopedic applications, represents an important regenerative option whose efficacy is being newly validated specifically for the TMJ field. Therefore, the significance of this study lies in its contribution to broadening the therapeutic spectrum for TMD; it expands the clinical evidence for new regenerative injection therapies and helps to diversify the treatment options available beyond conventional methods.

In this study, 77.3% of patients exhibited improved outcomes following repeated prolotherapy sessions. Most notably, significant improvements in VAS and MMO were observed after the first session (Fig. 2). The slight increase in VAS observed after the second session was not statistically significant, suggesting it likely represents normal clinical variability within the treatment course rather than a true adverse effect of the therapy. However, additional injections (1–2 sessions) and follow-up evaluations led to the resolution of subjective symptoms. Similar findings have been reported in orthopedic research, where a minimum of three injection sessions yielded the most effective results [32]. Given the nature of joint disorders, chronic pain often leads to restricted range of motion. Even after pain relief, soft tissue discomfort may persist during the rehabilitation phase due to underutilization of these tissues. Although additional prolotherapy sessions were administered during the follow-up period, this study suggests that conservative management without further interventions could also be effective. This study also revealed a statistically significant correlation between improvements in MMO and pain reduction (Fig. 3). Based on clinical experience, the authors observed that patients who actively engaged in rehabilitation exercises following prolotherapy tended to achieve better treatment outcomes. Therefore, clinicians should emphasize the importance of rehabilitation exercises and provide motivation to patients post-intervention to maximize therapeutic effects. Stabilization splint therapy did not significantly influence prolotherapy outcomes in this study. This may be attributed to the relatively passive nature of splint therapy compared to prolotherapy, which actively promotes tissue regeneration. While stabilization splint therapy remains a promising treatment option, its limitations in encouraging active rehabilitation may explain its lesser impact on outcomes in this context.

Among the 39 patients who presented with joint sounds before treatment, 23 patients (60%) experienced complete resolution of joint sounds following prolotherapy. Additionally, approximately half of these patients showed improvements in jaw deviation during mouth opening (Table 3). These findings suggest that prolotherapy may help stabilize the retrodiscal tissue or reduce local inflammation, potentially leading to improved joint mechanics and a reduction in symptoms associated with disc derangement. However, this study’s retrospective design posed limitations, as imaging techniques such as Magnetic Resonance Imaging (MRI) were not used to confirm changes in the position or structure of the articular disc. Although significant clinical improvements in MMO and pain scores were observed, further investigation is necessary to determine the precise tissue-level effects of prolotherapy. Future studies should adopt a prospective design incorporating advanced imaging modalities, such as MRI, to evaluate soft tissue changes before and after treatment. More fundamentally, the primary limitation of this study is the absence of a placebo or no-treatment control group. As a retrospective study, our findings are associative and not definitive evidence of causality. Consequently, the observed clinical improvements cannot be definitively attributed solely to the intervention, as the potential influences of natural recovery or a placebo effect cannot be excluded. Prospective randomized studies are needed to confirm these results. A further limitation of this study is the use of a non-standard diagnostic classification that did not clearly differentiate between arthrogenous and myogenous TMD. While we attempted to address this by comparing our “bone-related” and “soft-tissue-related” groups, we found no statistically significant difference in treatment outcomes. However, this result must be interpreted with caution. The “soft-tissue-related” group was inherently heterogeneous, which may have obscured potential differential effects of the therapy, and the small sample size of the osteoarthritis group limited the statistical power of this subgroup comparison. Future research should therefore prioritize the use of standardized diagnostic criteria, such as the DC/TMD, to enroll more homogeneous patient populations. Specifically, including a larger cohort of patients with osteoarthritis or other bony abnormalities could provide a more comprehensive understanding of prolotherapy’s therapeutic mechanisms and its long-term efficacy in addressing structural and functional aspects of TMD. Furthermore, the discrepancy in treatment intervals between the groups (at 1- to 2-week intervals for PDRN *vs*. tri-weekly for dextrose) is a significant confounder that makes it difficult to isolate the pharmacological effects of the agents from the effects of treatment frequency.

Finally, prolotherapy for the TMJ is an emerging field with expanding clinical applications, and the potential of new agents such as PDRN is being actively explored. Consequently, a key limitation is the current absence of clearly defined indications and optimized treatment protocols. Accordingly, this study aimed to explore potential indications and effective protocols for prolotherapy using PDRN and hypertonic dextrose, based on its clinical outcomes. Future research is necessary to establish specific indications based on the mechanistic characteristics of each agent and to develop a standardized protocol by optimizing the injection site, dosage, frequency, and interval.

## 5. Conclusions

Prolotherapy is an effective intervention for improving pain, jaw function, and joint stability in patients with refractory TMD. Both hypertonic dextrose and PDRN showed comparable efficacy, offering significant clinical improvements. These findings support prolotherapy as a minimally invasive alternative for TMD management, particularly for patients with persistent symptoms unresponsive to conventional therapies. Further studies are needed to elucidate long-term outcomes and refine treatment protocols.
